# Response to comment on “Oestrogen-induced angiogenesis and implantation contribute to the development of parasitic myomas after laparoscopic morcellation”

**DOI:** 10.1186/s12958-017-0270-5

**Published:** 2017-07-20

**Authors:** Ben-Shian Huang, Huann-Cheng Horng, Peng-Hui Wang, Muh-Hwa Yang, Yi-Jen Chen

**Affiliations:** 1 Department of Obstetrics and Gynaecology, Taipei Veterans General Hospital, No.201, Sec. 2, Shih-Pai Road, Taipei, 112 Taiwan; 2 Department of Obstetrics and Gynaecology, National Yang-Ming University Hospital, No.152, Xin-Min Road, Yilan, 260 Taiwan; 3 Department of Obstetrics and Gynaecology, School of Medicine, No.155, Sec.2, Li-Nong Street, Taipei, 112 Taiwan; 4 Institute of Clinical Medicine, National Yang-Ming University, No.155, Sec.2, Li-Nong Street, Taipei, 112 Taiwan; 5 Department of Obstetrics and Gynaecology, Cheng-Hsin General Hospital, No.45, Cheng Hsin St., Pei-Tou, Taipei Taiwan

## Abstract

According to the literature review, CO2 insufflation on parasitic myoma implantation is not well studied, and we concur that our study is related to “Morcellation-induced parasitic myomas.” We did not compare CO2 insufflation to non-insufflation in our study. The reason is the efficacy of gasless laparoscopic myomectomy and morcellation is not well established and this modality is seldom performed. Moreover, the effects of pneumoperitoneum on mesothelial cells and the role of the entire peritoneal cavity as a cofactor in adhesion formation have become well established, the role of CO2 insufflation in the establishment of parasitic myomas has not yet been studied. As such, more in-depth and well-designed studies for the role of CO2 insufflation are needed.

## Response

We appreciate the interest in our manuscript by Mynbaev et al. [1] but find the criticism debatable. We concur that the heterogeneity of myomas, even from the same uterus, can have different mutations and diverse features depending on their size and location (submucosal, intramural and subserosal). In addition, we also concur with another view of the myoma pseudo-capsule, which consists of a fibro-neuro-vascular network and initiates angiogenesis and vascularization in the myomas. In this study, we received Ethics approval and consent to participate as well as for publication from the patients in our original article (VGHIRB No 2014–10-002C for human tissue; IACUC 2014–119 for animal study). We only used the intramural type of myoma and the central part of myoma tissue, which reduced the issue of heterogeneity of uterine myomas. Moreover, before each xenograft procedure in our study, we mixed myoma specimens obtained from two patients. The myoma fragments were cultured with E2-containing medium, which provided consistency of the oestrogen effect before xenotransplantation to eliminate individual differences in systemic/local oestrogen levels. Uterine myoma is also known as an oestrogen-dependent disorder, and myomas from women in different phases might present different systemic/local oestrogen levels. In addition, the tissue fragments, which were 1–2 mm^3^ in size, were manipulated aseptically at room temperature; but a culture time of more than 4 h may be associated with lower viability of the implanted tissue fragments in the in vivo study [2, 3]. This xenograft mouse model has been established.

According to the literature review, CO_2_ insufflation on parasitic myoma implantation is not well studied, and we concur that our study is related to “Morcellation-induced parasitic myomas.” We did not compare CO2 insufflation to non-insufflation in our study. The reason is the efficacy of gasless laparoscopic myomectomy and morcellation is not well established and this modality is seldom performed [4, 5].

The pathophysiology of adhesion formation is traditionally considered a local phenomenon resulting from surgical trauma to the peritoneal surfaces, as well as involving the basal membrane, mesothelial cells, and sub-endothelial connective tissue. This leads to a local inflammatory reaction and a cascade of events, such as exudation, fibrin deposition, and capillary growth at the site of injury [6]. Moreover, postoperative adhesion increases with desiccation and with the duration and pressure of carbon dioxide (CO_2_) pneumoperitoneum by insufflation through a subnormal mesothelial partial O_2_ pressure. It increases if the pneumoperitoneum contains more than 10% O_2_ through a supernormal mesothelial partial O_2_ pressure and reactive O_2_ species (ROS) [7]. Evidence of decreased acute inflammation includes the prevention of desiccation by humidified gas, gentle tissue handling as evidenced by the decreasing adhesions during the learning curve, and a physiologic mesothelial partial O_2_ pressure of approximately 30 mmHg by adding 4% O_2_ and/or 10% nitrous oxide (N_2_O) to the CO_2_ pneumoperitoneum [8–10]. However, the above-mentioned postoperative adhesion mouse model was very different from that used in our study. Binda et al. have conducted excellent studies examining the association between artificial bipolar lesions and CO_2_ pneumoperitoneum by insufflation [7–10]. In the model we used, there was only a 1-cm wound created, and we avoided manipulations to minimize tissue injury during xenograft procedures. Pneumoperitoneum was simulated by a pneumoperitoneum needle (Surgineedle™, Covidien, US) inserted into the abdominal cavity. The CO_2_ insufflation pressure was 4 mmHg, and the duration of insufflation was 10 min [11].

Although the effects of pneumoperitoneum on mesothelial cells and the role of the entire peritoneal cavity as a cofactor in adhesion formation have become well established, the role of CO_2_ insufflation in the establishment of parasitic myomas has not yet been studied. As such, more in-depth and well-designed studies for the role of CO_2_ insufflation are needed.
